# TMPRSS2-ERG Fusion Identified by Comprehensive Genomic Profiling Reveals Prostatic Origin in Cancer of Unknown Primary: A Case Report

**DOI:** 10.7759/cureus.106438

**Published:** 2026-04-04

**Authors:** Koji Hikino, Wataru Naito, Shinichi Teshima, Yohei Miyagi, Akira Sawaki

**Affiliations:** 1 Department of Clinical Oncology, Shonan Kamakura General Hospital, Kamakura, JPN; 2 Department of Pathology, Shonan Kamakura General Hospital, Kamakura, JPN; 3 Division of Molecular Pathology and Genetics, Kanagawa Cancer Center Research Institute, Yokohama, JPN; 4 Department of Pathology, Kanagawa Cancer Center Research Institute, Yokohama, JPN

**Keywords:** cancer of unknown primary, comprehensive genomic profiling, gene fusion, lymph node metastasis, molecular diagnosis, prostate cancer, tissue of origin, tmprss2-erg fusion

## Abstract

Cancer of unknown primary (CUP) comprises a heterogeneous group of malignancies in which the primary site cannot be identified despite comprehensive evaluation. Patients in the unfavorable-risk group have limited therapeutic options and typically receive empirical chemotherapy, which is associated with poor outcomes. Comprehensive genomic profiling (CGP) has emerged as a valuable tool for identifying potential therapeutic targets in advanced or rare solid tumors, including cancer of unknown primary. However, the proportion of actionable alterations remains limited, and genomic findings that do not directly inform treatment selection have received relatively little attention, despite their potential relevance for identifying the tissue of origin. We report the case of a 79-year-old man presenting with multiple lymph node metastases from an unknown primary and clinically localized prostate cancer. Comprehensive genomic profiling was performed on a metastatic lymph node specimen. Although no alterations with immediate therapeutic relevance were identified, a *TMPRSS2-ERG* fusion was detected, supporting a prostatic origin. This case highlights that comprehensive genomic profiling may contribute not only to therapeutic decision-making but also to the identification of the tissue of origin in diagnostically challenging cases.

## Introduction

Cancer of unknown primary (CUP) refers to a clinical condition characterized by metastatic disease in which the primary site remains unidentified after a thorough diagnostic workup, including imaging, endoscopy, and histopathological assessment. CUP generally carries a poor prognosis, particularly in the unfavorable-risk group, which is defined by the absence of clinically favorable subsets and is associated with a median overall survival of approximately 6-9 months [1]. In contrast, selected favorable subsets, such as women with peritoneal carcinomatosis consistent with serous carcinoma or patients with isolated cervical lymph node metastases of squamous cell carcinoma, may achieve substantially longer survival when treated according to the presumed tissue of origin [2]. Therefore, the accurate identification of biologically favorable subsets is clinically crucial.

For patients in the unfavorable-risk group, platinum-based combination chemotherapy remains the standard treatment, although therapeutic outcomes remain limited. Given the modest efficacy of cytotoxic chemotherapy, immune checkpoint inhibitors have been investigated as alternative therapeutic strategies in patients with CUP. In Japan, a phase II study demonstrated the promising clinical activity of nivolumab in patients with CUP, suggesting its potential as a viable clinical option in selected cases [3]. Owing to the diagnostic and therapeutic challenges of CUP, comprehensive genomic profiling (CGP) has become increasingly integrated into clinical practice for advanced or rare solid tumors, including CUP, not only to identify potentially targetable genomic alterations but also to provide molecular clues regarding the tissue of origin. While the clinical utility of CGP is often discussed in terms of targetable alterations, its diagnostic value in refining tumor classification may be equally important.

In the present case, CGP identified *TMPRSS2-ERG* fusion. Although this alteration is not directly linked to approved targeted therapies, it represents a molecular alteration strongly associated with prostate cancer. This case highlights the importance of interpreting CGP results beyond therapeutic actionability and underscores the potential role of organ-specific genomic alterations in guiding diagnostic reclassification and the refinement of tissue-of-origin assessment in CUP.

## Case presentation

**Figure 1:**
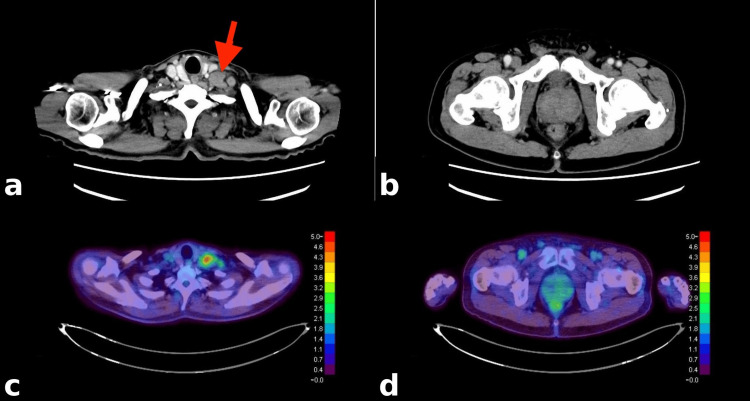
Pretreatment CT and FDG-PET images. (a and c) Contrast-enhanced CT and FDG-PET images of a metastatic cervical lymph node (red arrow), demonstrating increased FDG uptake (SUVmax: 5.3). (b and d) Contrast-enhanced CT and FDG-PET images of the prostate gland, showing no abnormal FDG uptake. CT, computed tomography; FDG-PET, 18F-fluorodeoxyglucose positron emission tomography; SUVmax, maximum standardized uptake value

A 79-year-old man was referred to our hospital in June 2022 with a two-week history of fever and palpable lymphadenopathy in the cervical and inguinal regions. His medical history was notable for benign prostatic hyperplasia, and his family history included pancreatic cancer. On physical examination, firm, immobile lymph nodes measuring approximately 10 mm were palpable in the left cervical and left inguinal regions. Laboratory testing at presentation revealed a slightly elevated prostate-specific antigen (PSA) level, while carcinoembryonic antigen and other routine laboratory parameters were within normal limits. Contrast-enhanced computed tomography (CT) from the neck to the pelvis demonstrated multiple enlarged lymph nodes in the left cervical, supraclavicular, mediastinal, and para-aortic regions (Figure 1a, 1b).

**Figure 2:**
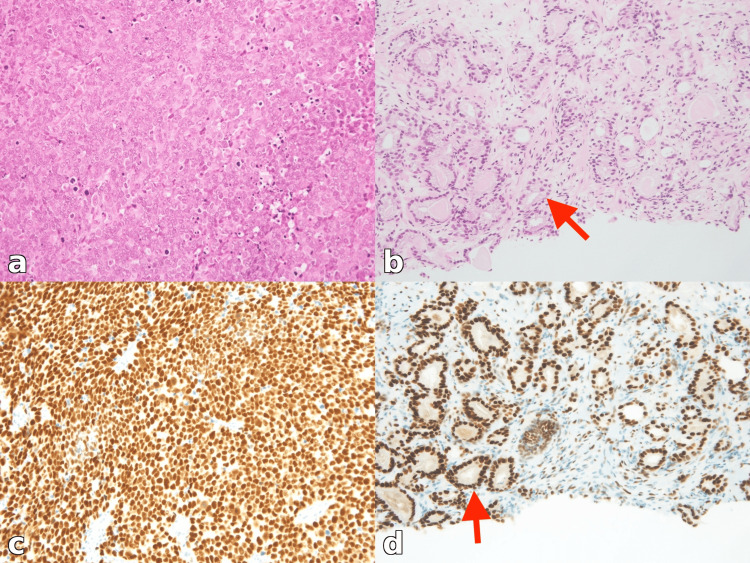
Histologic features of the metastatic lymph node (a and c) and the prostate biopsy specimen (b and d). (a) H&E staining of the lymph node shows poorly differentiated carcinoma with large atypical tumor cells, nuclear pleomorphism, and frequent mitotic figures. (b) H&E staining of the prostate biopsy demonstrates well-differentiated adenocarcinoma (red arrow). (c) The AR IHC of the lymph node shows strong nuclear staining. (d) The AR IHC of the prostate biopsy demonstrates strong nuclear staining (red arrow). (a-d) Original magnification: ×200. H&E, hematoxylin and eosin; AR, androgen receptor; IHC, immunohistochemistry

No definitive primary lesion was identified, except for mild prostate enlargement. Upper and lower gastrointestinal endoscopy showed no significant abnormalities. 18F-Fluorodeoxyglucose positron emission tomography (FDG-PET) revealed hypermetabolic activity in multiple lymph nodes without evidence of a primary tumor, including the prostate (Figure 1c, 1d). An excisional biopsy of a left supraclavicular lymph node was performed. Histopathological examination with hematoxylin and eosin staining demonstrated poorly differentiated carcinoma (Figure 2a).

Immunohistochemistry (IHC) showed positivity for AE1/AE3 and negativity for TTF-1, napsin A, p40, CK7, CK20, and CD56. The Ki-67 labeling index exceeded 80%. Based on a diagnosis of CUP after a comprehensive diagnostic evaluation, nivolumab (240 mg every two weeks) was initiated in July 2022.

**Figure 3:**
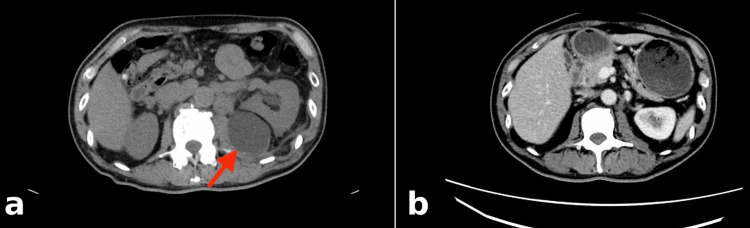
CT images of the retroperitoneal mass before and after degarelix treatment. (a) Metastatic mass before the initiation of degarelix therapy (red arrow). (b) Four weeks after the initiation of degarelix, the mass demonstrates a marked reduction in size. CT: computed tomography

After three cycles, follow-up CT demonstrated disease progression, including a newly detected lesion in the left retroperitoneal region. Nivolumab was discontinued. CGP was performed using the FoundationOne CDx assay (Foundation Medicine, Cambridge, MA) on the lymph node specimen. Given the gradually increasing PSA levels, a prostate biopsy was performed and revealed well-differentiated adenocarcinoma (Gleason score: 3+4) (Figure 2b). CGP identified *TMPRSS2-ERG* fusion, microsatellite stability, and a low tumor mutational burden (1.21 mutations/megabases {Mb}). Additional alterations included *NF1* splice-site mutation (7063-1G>T), *NF1* p.V1182F, and *STK11* truncating mutation (p.C210*); no targetable alterations were identified. To evaluate the potential relationship between the lymph node metastases and prostate carcinoma, androgen receptor (AR) IHC was performed on both the lymph node and prostate specimens. Strong nuclear AR expression was observed in both specimens (Figure 2c, 2d). Androgen deprivation therapy with degarelix acetate was initiated in September 2022. One month later, the PSA level markedly decreased (Table 1), and follow-up CT demonstrated the regression of multiple lymph node metastases, as well as a reduction of the retroperitoneal mass (Figure 3a, 3b).

**Table 1:** Time course of serum PSA levels. PSA levels were measured at each time point during the clinical course. The timing of each measurement is shown in relation to treatment initiation. Degarelix acetate was initiated after the third measurement. PSA: prostate-specific antigen

Time from treatment start	Clinical time point	PSA (ng/mL)
Before initiation	At the first visit	9.5
Before initiation	Two months later	19.5
Day 0	At treatment initiation (baseline)	63.9
Week 4	Four weeks after degarelix initiation	1.9
Reference range		<4.0

## Discussion

Clinically, the case presented a diagnostic discrepancy: widespread multistation lymph node metastases without an identifiable primary site, alongside localized prostate cancer. The distribution of lymphadenopathy raised suspicion for a non-prostatic primary. In addition, the PSA level at presentation and the degree of FDG uptake in the prostate were not consistent with the extent and distribution of nodal disease. Therefore, a CGP of a metastatic lymph node was undertaken to refine the presumed tissue of origin beyond clinicopathological assessment.

Pathologically, the metastatic lymph node showed a poorly differentiated carcinoma with epithelial differentiation; however, an extensive IHC evaluation did not identify a lineage-specific profile that would reliably indicate the primary site (see Case Presentation). Such non-informative or equivocal immunophenotypes are not uncommon in CUP and can limit tissue of origin estimation based on histology alone. When clinicopathological assessment remains inconclusive in CUP, organ-specific markers guided by the overall clinical presentation may add diagnostic value. In this case, the concurrent prostatic lesion supported the consideration of prostatic markers. Accordingly, because the primary site could not be established with certainty based on clinicopathological findings, CGP was performed to clarify the tissue of origin. Such histologic discordance between a poorly differentiated metastatic lesion and a well-differentiated primary tumor may reflect tumor heterogeneity or clonal evolution during metastatic progression.

The CGP analysis identified *TMPRSS2-ERG* fusion, a well-established and highly characteristic alteration in prostate cancer [4]. *TMPRSS2-ERG* fusion is among the most common gene fusions in prostate tumors, reported in a substantial proportion of cases [5]. In the setting of CUP, particularly when histology and immunophenotyping are non-informative, the detection of *TMPRSS2-ERG* fusion can provide strong molecular support for a prostatic tissue of origin. In this case, the genomic finding was also consistent with the AR-positive immunophenotype observed in the metastatic lesion, further supporting a prostatic origin. Importantly, this case illustrates that the clinical value of CGP extends beyond identifying actionable targets to include genomic clues that may assist in tissue of origin estimation and support diagnostic decision-making. In addition to *TMPRSS2-ERG* fusion, co-occurring alterations in *NF1* and *STK11* were identified. Although these alterations were not directly actionable in this case, both genes are involved in key oncogenic pathways. *NF1* loss has been associated with the dysregulation of RAS signaling, whereas *STK11* encodes *LKB1*, a central regulator of adenosine monophosphate-activated protein kinase (AMPK)-related cellular metabolism and growth control [6,7]. *STK11* alterations have also been implicated in tumor progression and metabolic reprogramming across multiple cancer types. However, the biological and clinical significance of these alterations in the context of prostate cancer or CUP remains incompletely understood. Therefore, their contribution to tumor behavior in this case remains uncertain.

Real-world Japanese data and the Cancer of Unknown Primary Site Consortium (CUPISCO) study demonstrate that actionable alterations are identified in only a subset of patients undergoing CGP [8,9]. Thus, the clinical value of CGP should not be judged solely by therapeutic actionability. In cases where clinicopathological evaluation is inconclusive, lineage-associated genomic alterations may help clarify the tissue of origin. In this patient, the detection of the *TMPRSS2-ERG* fusion provided strong molecular evidence for a prostatic origin, which contributed to appropriate clinical management.

However, several limitations should be acknowledged. This is a single case report, and the generalizability of these findings remains uncertain. In addition, no functional validation of the detected fusion was performed. The further accumulation of similar cases may help to better define the diagnostic role of CGP in CUP.

## Conclusions

At presentation, the disproportionately low PSA level and the histopathological discordance between the metastatic lymph node and the prostate lesion raised the possibility of two concurrent malignancies. However, the CGP of the metastatic lymph node identified a *TMPRSS2-ERG* fusion, an alteration strongly associated with prostate cancer, thereby establishing a prostatic origin for the metastasis. This case highlights that in CUP, CGP should be interpreted not only for therapeutic actionability but also for its potential to inform the tissue of origin. Organ-specific genomic alterations, such as *TMPRSS2-ERG* fusion, may therefore provide important diagnostic clues in the evaluation of CUP.
